# Health Tract, No. 242

**Published:** 1866-06

**Authors:** 


From the Boston Watchman and Reflector, by Dr. W.W. Hall, M.D.

HEALTH TRACT, NO. 242.

### EMERGENCIES.

If any thing swallowed by mistake causes an intense burning in the throat, it is probably a "corrosive" poison, that is, destroys the textures with which it comes in contact, send for a physician. Meanwhile swallow instantly half a glass of sweet or of sperm oil ,or melted butter, or lard, whichever is most convenient to use, and then, within five minutes, half a pint of water in which has been stirred a tea spoonful each of common ground table mustard and salt.

When a poison has been swallowed which has no special effect on the throat, but causes sickness at the stomach, faintness, drowsiness, stupor, or any other strikingly unusual or unnatural feeling, swallow instantly the whites of two or three eggs, and, as quickly as can be prepared, half a pint of coffee made thus: On a tea-cupful of ground coffee pour half a pint of boiling water. Stir into it the white of an egg. After allowing it to rest a minute or two, pour the liquid into a cold cup, and when it is not too hot, drink it. Then, within five minutes, pour a glass of water on a tablespoonful each of ground mustard and table salt, stir and drink it at once, so as to prevent the mustard from settling on the bottom of the glass. The egg in the stomach more instantly antagonizes a large number of poisons than any other known substance; the coffee acts thus on the next largest number of poisons; while the mustard mixture relieves the stomach of the whole of its contents by vomiting more instantly and safety than any other familiar compound. This prescription has the incalculable advantages of being always at hand; its constituents are familiar to every one; and are perfectly harmless in any quantity likely to be taken.

If a person faints, place him on his back and let him alone until he "comes to," for the heart ceased to beat with force enough to carry purified blood to the head, and when it begins to beat again, it requires less power to propel the blood there when the person is lying down than when he is in a sitting or standing posture. Cutting garments, dashing cold water, or pouring brandy down the throat are unnecessary interferences.

If any part of the body is scalded or burned, put it instantly under cold water, and let it remain there until the physician arrives. The cessation from pain is nearly always instantaneous. If a physician cannot be obtained within an hour or two, apply a handful of dry flour to the burned part until it is covered a quarter of an inch or more deep, so as effectually to keep from it the air which causes the pain of a burn. Tie a piece of linen or cotton cloth lightly around, if it is possible to do so, and let the patient go to sleep. If the burn is very severe let him live wholly on coarse bread and fruits in any shape or form, but not sweetened. If the burn is not deep, there will be no suffering; healing will commence in a few hours, and, as new skin forms, the flour will drop off, or may be moistened with warm water and carefully removed. This is the best, safest and least painful treatment for ordinary burns and scalds.

